# Case of Imperforate Anus, with Deformity of the Organs of Generation

**Published:** 1871-01

**Authors:** Moses Barrett

**Affiliations:** Milwaukee, Wis.


Article VI. — *Case of Imperforate Anus, with Deformity of the Organs of Generation.* By Moses Barrett, M.D., Milwaukee, Wis.

Mrs. K., a German woman, was delivered on the morning of Sept 12, 1870, of a full grown child. She was attended by a woman, who discovered, on dressing the child, an unusual appearance of the genital organs, and a complete closure of the anus. I was called about six hours after the birth of the child. It was of large size, well developed, and to all appearance natural with the exception before mentioned. The abdomen was very much distended, particularly the lower part up to the umbilicus, and very hard, giving a flat sound on percussion. The attendant stated that more difficulty was experienced in delivering the body than the head. There was no appearance of any anus, or fissure along the perineum. There was a fold of the integument just below the pubis which well represented the external labia, from between which there projected a penis-like organ, about two inches in length, from the extremity of which urine was continually oozing out. There were no cavernous bodies or glans, but simply a pendulous mass of integument, oval in shape, the longest diameter being about half an inch. Along the upper part a round cord could be felt, which proved to be the urethra, terminating at the extremity, with the integument folded about after the form of a prepuce. So much did this organ resemble a penis, that the child was called a male, and baptized as such.

There was no opening between the labia, and the whole perineum was uniformly thick and unyielding. Hoping to find the distended rectum, I made an incision through the integument along the mesian line, from the point of the coccyx about one inch and a half. No signs of the rectum appearing, the dissection was carefully continued through the sphincter muscle, and along the curve of the sacrum over two inches. As neither rectum nor vagina could be discovered, the opening was enlarged so as to admit of the introduction of the little finger, which was passed up nearly the whole length, when a sac of fluid was distinctly felt. This was punctured with a bistoury and about twenty ounces of fluid passed off. At first I supposed I had punctured the bladder, but upon examination the fluid was ascertained to be serum. The child was immediately relieved, and the tumefaction of the abdomen nearly disappeared. No further attempt was made to reach the intestine, as it was evident a fatal result must inevitably follow.

I saw the child again on the third day, found that it had taken nourishment, slept well, and urinated freely. During the first twenty-four hours after the operation serum continued to flow from the opening. I passed a common director up the passage formed, which entered the whole length without any obstruction. The child died on the morning of the third day.

*Autopsy.* On opening the abdomen, there were no adhesions, and no fluid was found in the cavity of the peritoneum. The stomach and small intestines were natural, and the large intestine also, except about six inches of the lower extremity, which was enlarged about twice the natural diameter. But the most singular object presented, was a bladder-like organ extending up to the umbilicus, which proved to be an enlarged *uterus*, with the fallopian tubes enlarged and sacculated—the right one of the size of a hen's egg. The bladder was situated in front, of natural size. The enlarged portion of the colon extended transversely behind the uterus. On separating the reflected peritoneum the intestine was found to terminate in a *cul-de-sac*, just as it entered the pelvis, and was firmly attached to the posterior surface of the uterus, near the lower extremity. There was no communication from the intestine to the cavity of the uterus, and the interior surface presented much the appearance of the natural rectum at the sphincter, with consecutive folds of mucous membrane and a central point or cicatrix. There seemed to be enough of intestine originally formed to reach the anus had it not been prevented from entering the pelvis by the enlarged uterus, or possibly, drawn up with that organ as it became distended with the serum.

The uterus measured over five inches in length and four and a half in breadth. There was no neck, but a small round opening at the lower part, resembling the os. I found I had punctured the organ just posterior to this point. The ovaries were found of natural size. Careful examination was made for testes, but none were found.

The case presents one of the most singular malformations on record. The abnormal development undoubtedly commenced in the uterus very early in the period of fœtal life, and the closure of the perineum was the natural result. But what is remarkable, was the prolongation of the urethra with folds of the integument to the extent found. Nothing else had the slightest appearance of a male child.

